# Affective Teacher—Student Relationships and Students' Externalizing Behavior Problems: A Meta-Analysis

**DOI:** 10.3389/fpsyg.2016.01311

**Published:** 2016-08-30

**Authors:** Hao Lei, Yunhuo Cui, Ming Ming Chiu

**Affiliations:** ^1^Institute of Curriculum and Instruction, East China Normal UniversityShanghai, China; ^2^College of Education, Purdue UniversityWest Lafayette, IN, USA

**Keywords:** affective teacher—student relationships, externalizing behavior problems, meta-analysis, students

## Abstract

This meta-analysis of 57 primary studies with 73,933 students shows strong links between affective teacher—student relationships (TSRs) and students' externalizing behavior problems (EBPs). Moreover, students' culture, age, gender, and the report types of EBPs moderated these effects. The negative correlation between positive indicators of affective TSRs and students' EBPs was stronger (a) among Western students than Eastern ones, (b) for students in the lower grades of primary school than for other students, (c) when rated by teachers or parents than by students or peers, and (d) among females than among males. In contrast, the positive correlation between negative indicators of affective TSRs and students' EBPs was stronger (a) among Eastern students than Western ones, (b) for students in the higher grades of primary school than for other students, and (c) when rated by students or peers than by teachers or parents.

## Introduction

Behavior problems occur when an individual violates social norms or rules of behavior (*social maladjustment*), leading to adverse effects and possibly behaviors that are harmful to himself or herself, others or even society (Zhang, 1999).

Over the past decade, researched on behavior problems have attracted the attention of an increasing number of psychology, education, sociology, and even psychopathology experts. Many researchers have explored the influence of school climate, parenting style, child–parent relationships, and family function on students' behavior problems (Haynes et al., 1997; Aunola and Nurmi, 2005; Pettit and Arsiwalla, 2008; Thornton et al., 2008). In particular, researchers have examined the links between teacher–student relationships (TSRs) and students' behavior problems (Vick, 2008; Ewing, 2009; Leflot et al., 2011; Spilt et al., 2012c; De Laet et al., 2014). Many theoretical and empirical studies have yield varied conclusions (Gest et al., 2005; Palermo et al., 2007; Doumen et al., 2009a). Nevertheless, the scope of problem behaviors includes many factors with different orientations and natures. This has led researchers to neglect communication with each other and avoid comparisons of their results.

Some researchers maintain that behavior problems should be classified as externalizing behavior problems (EBPs) vs. internalizing behavior problems (IBPs; Achenbach and Dumenci, 2001). EBPs are individual reflections regarding an external environment with negative external behaviors (Liu, 2004). Researchers have adopted different standards for classifying EBPs. For example, consider two dimensions: openness–concealment and destructive–non-destructive. Loeber et al. (2000) believe that EBPs should be divided into aggression, agonistic behavior, property damage, and reputation infringement. Based on individual behaviors, Cai and Zhou (2006) argued that EBPs should be divided into hyperactivity, aggression, and conduct problems. In contrast, IBPs are negative moods and emotions that lead to emotional disorder, including depression, anxiety, withdrawal, and guilt (Zanh–Waxler et al., 2000).

Highlighting these definitions of behavior problems clarifies various concepts' theoretical boundaries that determine the nature, direction, and veracity of research inquiries. According to these definitions, EBPs tend to be explicit and more destructive than IBPs. More importantly, several studies found that the correlation between affective TSRs and EBPs was stronger than that the correlation between affective TSRs and IBPs (Zhang and Sun, 2011; Gyllborg, 2013). Therefore, this study used a meta-analysis to explore the link between affective TSRs and students' EBPs, and excluded IBPs.

Researchers used different indicators of EBPs. For example, Achenbach (1991) developed the *Achenbach child-behavior checklist* (CBCL), according to which EBPs included delinquent behavior and aggressive behavior; Reynolds (2004) developed the *Behavior Assessment System for Children–Teacher Rating Scales for Children (BASC–TRS)*, in which EBPs include hyperactivity, aggression, and conduct problems. Thus, in accordance with these previous studies, this study will consider delinquency, aggression, hyperactivity, and conduct problems as indicators of EBPs.

TSRs are an important component of interpersonal communication ability and social adaptability. This study focuses on a specific subset of TSRs, namely affective TSRs; this choice was inspired by Cornelius-White's findings that the affective variables “empathy” and “warmth” are strongly associated with student outcomes (Cornelius-White, 2007). Roorda et al. (2011) considered both positive and negative indicators of affective TSRs. Specifically, positive indicators of affective TSRs comprise closeness, support, liking, warmth, and trust. In contrast, negative indicators comprise conflict, anger, and dislike. Although some argue that dependency is a component of affective TSRs (Pianta, 2001; Fraire et al., 2013; Settanni et al., 2015), other studies using multiple methods to examine relationship quality questioned the validity of dependency as a measure of dyadic relationship quality (Doumen et al., 2009b; Roorda et al., 2011); thus, dependency was excluded from this study.

According to *stage—environment fit theory*, individual development requires an interpersonal relationship that has trust, support, caring, self-expression, self-choice, and self-determination; in cases where. A teacher who did not provide these interpersonal relationships and opportunities created an environmental mismatch with individual development, thus leading to students showing EBPs (Wang, 2009; van Lier et al., 2012; Loukas et al., 2013). Moreover, many empirical studies have found that positive indicators of TSRs were negatively correlated with students' EBPs (Gest et al., 2005; Koomen et al., 2012; Spilt et al., 2012a; Thijs et al., 2012) while negative indicators of affective TSRs were positively correlated with students' EBPs (Doumen et al., 2009a; Spilt et al., 2012a). However, correlations varied across studies. To resolve this issue, several researchers have summarized research results with reviews (Baker et al., 2008; Nurmi, 2012), but these studies only partly verified the phenomena. Their limitations include convenience samples, various sample sizes, or ignoring moderators, which led to inconsistencies and low reliability. Therefore, a meta-analysis is needed to determine the link between affective TSRs and students' EBPs.

Our review of past empirical studies showed that many effect sizes were heterogeneous, suggesting that moderating factors might account for different links between affective TSRs and students' EBPs. Thus, we hypothesized that one or more variables may moderate the effect sizes of the correlation between affective TSRs and students' EBPs, such as differences in students' cultures, ages, genders, and the report types of EBPs.

First, we examine whether students' culture (as a latent variable) moderates the link between affective TSRs and students' EBPs (Chang et al., 2007; Roorda et al., 2014). Several studies suggest that culture influences the link between affective TSRs and students' EBPs (e.g., closeness and EBP, and conflict and EBPs). Baker (2006) found a moderate correlation between closeness and students' EBPs among Western students; however, Ly (2013), whose sample included Eastern students, found a weak correlation between the two factors. Many studies found a strong correlation between conflict and students' EBPs among Western students (Doumen et al., 2008, 2009a; Ly, 2013); however, Fu (2014), whose sample included Eastern students, found moderate correlation between the two factors. Thus, in accordance with these findings, this study tests whether the correlation between positive indicators of affective TSRs and students' EBPs for Western students is stronger than that for Eastern students, and whether the correlation between positive indicators of affective TSRs and students' EBPs for Western students is weaker than for Eastern students.

Second, as the level of affective TSRs and students' EBPs might differ as a function of students' age (Zhang et al., 2008), we test whether students' age moderates the link between affective TSRs and students' EBPs. Differences in age have been found in the correlations between affective TSRs and students' EBPs. For example, previous studies indicated that positive indicators for affective TSRs and students' EBPs varied among students in kindergarten lower primary grades (LPG), and higher primary grades (Silver et al., 2005; HPG, Kuhns, 2008; Stewart and Suldo, 2011). In contrast negative indicators of affective TSRs and students' EBPs among kindergarten, LPG, and HPG students all showed similar phenomenon (Ezzell et al., 2000; Pianta and Stuhlman, 2004; Vick, 2008; Troop-Gordon and Kopp, 2011; Rudasill et al., 2013). Based on these findings, we expect age to moderate the link between affective TSRs and students' EBPs.

Third, we examine whether the report type of EBPs (as a latent variable) moderates the link between affective TSRs and students' EBPs. Raters with different ages, standpoints, values, and degrees of understanding a student might rate his or her EBPs inconsistently (Van Lier et al., 2005; Ladd, 2006). Moreover, several studies have found that different raters might account for the lack of coherence in research on the link between affective TSRs and students' EBPs. For example, some previous studies have relied on EBPs rated by students, which were only weakly related to positive indicators of affective TSRs (Troop-Gordon and Kopp, 2011; Li et al., 2012) while other studies found that student EBPs rated by teachers were moderately related to both positive indicators of affective TSRs (Colwell and Lindsey, 2003; Shin and Kim, 2008) and negative indicators of affective TSRs (White and Renk, 2012; Ly, 2013; Skalická et al., 2015). In contrast, other researchers found that student EBPs rated by teachers were strongly related to negative indicators of affective TSRs (Palermo et al., 2007; Fowler et al., 2008; Stipek and Miles, 2008). Thus, in accordance with these findings, we test whether *Report type of EBPs* moderate the link between affective TSRs and students' EBPs.

Fourth, we test whether gender (as a latent variable) moderates the link between affective TSRs and students' EBPs. Female students tend to have more affective TSRs than male students do (Spilt et al., 2012b), and male students tend to develop more EBPs than female students do (Hill et al., 2006). As a result, gender might influence the correlation between positive or negative indicators of affective TSRs and students' EBPs. Several empirical studies have showed gender differences in the link between indicators of affective TSRs and students' EBPs, such as closeness, support, and warmth (Ostrov and Crick, 2007; Spilt et al., 2012a; Thijs et al., 2012). Hence, these findings suggest that gender moderates the link between affective TSRs and students' EBPs.

### Study purpose

The current study models the link between affective TSRs and students' EBPs using meta-analysis. Specifically, this study (a) estimates the effect sizes of correlations between affective TSRs and students' EBPs and (b) tests whether the links between affective TSRs and students' EBPs are moderated by culture, age, report type of EBP, or gender.

## Methods

### Literature search

To identify studies on affective TSRs and students' EBPs, we systematically searched the literature from January 2000 to March 2016 in electronic databases, including ProQuest Dissertations, Web of Science, Google Scholar, Springer, Taylor & Francis, EBSCO, PsycINFO, and Elsevier SDOL. Indexed keywords primarily included terms reflecting affective TSRs (relationship(s), closeness, warmth, support, empathy, trust, sensitivity, conflict, negativity, and anger) and students' EBPs (behavior problems, externalizing, aggression, conduct problem, hyperactivity, and oppositional). When articles could not be found online, we obtained full-text versions of articles from libraries. All articles obtained were written in English. We used inclusion and exclusion criteria to analyze and filter the collected studies.

### Literature exclusion criteria

We included articles based on the following criteria: (a) tested the relation between affective TSRs and students' EBPs; (b) measured affective TSRs, including closeness, warmth, support, empathy, trust, sensitivity, conflict, negativity, or anger; (c) measured EBPs, including behavior problems, externalizing, aggression, conduct problem, hyperactivity, oppositional, or other indicators, (d) included an explicit sample size, and (e) explicitly reported the Pearson product-moment correlation coefficient (or a *t* or *F*-value that could be transformed into *r*). Table 1 summarizes the studies included in the Meta-Analysis.

**Table 1:** **Studies included in the meta-analysis**.

**Author (year)**	**Sample^a^**	**N^b^**	**Affective indicator**	**Report type (EBPs)**	**Male (%)^b^**
Baker, 2006	Western, mixed	1310	Conflict, closeness	Teacher	0.480
Colwell and Lindsey, 2003	Western, kindergarten	27 and 20	Positive emotions, negative emotions	Teacher	1.000 and 0.000
De Laet et al., 2014	Western, higher grades	586	Conflict, closeness	Peer	0.471
Doumen et al., 2008	Western, kindergarten	176	Conflict	Teacher	0.480
Doumen et al., 2009a	Western, kindergarten	212	Conflict	Teacher	0.481
Ewing, 2009	Western, higher grades	333 and 349	Conflict, closeness	Teacher	1.000 and 0.000
Ewing and Taylor, 2009	Western, kindergarten	158 and 143	Conflict, closeness	Teacher	1.000 and 0.000
Ezzell et al., 2000	Western, higher grades	37	Support	Parents	0.460
Fowler et al., 2008	Western, mixed	230	Conflict, closeness	Teacher	0.552
Fu, 2014	Western, kindergarten	1161 and 1100	Conflict, closeness	Teacher	No reports
Gest et al., 2005	Western, higher grades	383	Conflict, support	Peer, teacher	0.548
Gyllborg, 2013	Western, higher grades	53 and 63	Conflict, closeness	Student, teacher	1.000 and 0.000
Henricsson and Rydell, 2004	Western, lower grades	95	Anger, conflicts, closeness	Teacher	0.520
Howes, 2000	Western, lower grades	307	Conflict, closeness	Teacher	0.505
Howes et al., 2000	Western, kindergarten	357	Conflicts, closeness	Teacher	0.510
Hughes and Kwok, 2006	Western, lower grades	415	Conflicts, support	Peer, teacher	0.522
Hughes et al., 2001	Western, higher grades	993	Conflicts, support	Teacher	0.500
Koomen et al., 2012	Western, mixed	2335	Conflicts, closeness	Parents, teacher	0.488
Ladd and Burgess, 2001	Western, kindergarten, lower grades	385	Support	Peer, teacher	0.501
Lee and Bierman, 2015	Western, kindergarten, lower grades	164	Closeness	Teacher	0.440
Leflot et al., 2011	Western, lower grades	570	Support	Peer, teacher	0.495
Li et al., 2012	Western, lower grades	709	Support	Peer, student, teacher	0.533
Luckner and Pianta, 2011	Western, higher grades	894	Support	Peer	0.502
Ly, 2013	Eastern, lower grades	258	Conflict, closeness	Student, teacher	0.529
Murray and Murray, 2004	Western, higher grades	127	Conflict, closeness	Teacher	0.510
Murray and Zvoch, 2010	Western, mixed	171	Trust	Student, teacher	0.400
Murray and Zvoch, 2011	Western, higher grades	193	Conflict, closeness, trust	Student, teacher	0.435
Ostrov and Crick, 2007	Western, kindergarten	116	Conflict	Teacher	0.466
Palermo et al., 2007	Western, kindergarten	95	Conflict, closeness	Teacher	0.520
Pianta and Stuhlman, 2004	Western, lower grades	490	Conflict, closeness	Teacher	0.510
Roorda et al., 2014	Western, kindergarten	175	Conflict, closeness	Teacher	1.000
Rucinski, 2015	Western, higher grades	526	Conflict, closeness	Student, teacher	0.462
Rudasill et al., 2013	Western, lower grades	1363	Conflict, closeness	Parent	0.520
Rueger et al., 2008	Western, middle school	108 and 138	Support	Parent	1.000 and 0.000
Runions, 2014	Western, kindergarten, lower grades	749	Conflict, closeness	Teacher	0.480
Runions et al., 2014	Western, kindergarten	374	Conflict, closeness	Teacher	No reports
Runions and Shaw, 2013	Western, kindergarten	377	Conflict, closeness	Teacher	0.499
Sette et al., 2013	Western, kindergarten	88	Conflict, closeness	Teacher	0.523
Shin and Kim, 2008	Eastern, kindergarten	297	Conflict, closeness	Teacher	0.559
Silver et al., 2010	Western, kindergarten	241 and 283	Conflict, closeness	Parent	0.485 and 0.498
Silver et al., 2005	Western, kindergarten	283	Conflict, closeness	teacher	0.498
Skalická et al., 2015	Western, lower grades	981	Conflict, closeness	Parent, teacher	0.500
Solheim et al., 2011	Western, kindergarten	925	Conflict, closeness	Teacher	0.505
Spilt et al., 2012a	Western, lower grades	350 and 307	Conflict, warmth	Teacher	1.000 and 0.000
Spilt et al., 2012b	Western, kindergarten	188	Conflict, closeness, sensitivity	Teacher	0.553
Spilt et al., 2010	Western, kindergarten	150	Conflict, closeness, warmth	Student, teacher	0.540
Stewart and Suldo, 2011	Western, middle school	381	Support	Student	0.395
Stipek and Miles, 2008	Western, kindergarten, lower grades	301,330, 328, and 280	Conflict	Teacher	0.502, 0.491, 0.494, and 0.489
Suldo et al., 2012	Western, High school	415	Relationships	Teacher	0.400
Thijs et al., 2012	Western, higher grades	230	Conflict, closeness	Teacher	0.496
Troop-Gordon and Kopp, 2011	Western, lower grades	410	Conflict, closeness	Student	0.471
Vick, 2008	Western, kindergarten	100	Conflict, closeness	Teacher	0.460
Chang et al., 2007	Eastern, higher grades	730 and 635	Like	Student	1.000 and 0.000
Wang et al., 2015	Western, middle school	435	Caring	Student	0.568
White and Renk, 2012	Western, higher grades	206	Support	Student	0.510
Wolfson, 2009	Western, lower grades	96	Conflict, closeness	Teacher	0.490
Zhang and Sun, 2011	Eastern, lower grades	105	Conflict, closeness	Teacher	0.475

a Lower grades, lower grades of primary school, higher grades, higher grades of primary school

b Multiple numbers indicate multiple samples and the proportion of boys in each sample.

### Coding study

To facilitate meta-analysis, feature coding was conducted on 57 articles. We considered the following variables: author(s) and publication year, proportion of male students, age, indicators of affective TSRs, indicators of EBPs, number of students, and *r*. The following criteria guided the coding procedure: (a) effect sizes of each independent sample were encoded based on an independent sample, and effect sizes were separately encoded if a study had several independent samples; (b) if a study reported a correlation between affective TSRs and EBPs many times, the mean value was instead of effect sizes; (c) if an independent sample provided effect sizes (expressed as r) for sample characteristics such as gender, the results for the two genders were coded separately; (d) if a study reported not only a correlation between a total of EBPs and affective TSRs but also a correlation between the dimensions of EBPs and affective TSRs, we only coded the former; (e) if a study reported a correlation between different indicators of affective TSRs and EBPs, we coded these separately; and (f) if a study reported a correlation between indicators of affective TSRs and different indicators of EBPs, we coded these separately.

When coding was complete, based on principles of meta-analysis (Lipsey and Wilson, 2001), effect sizes between affective TSRs and students' EBPs were calculated for each sample. Then, we test whether the links between affective TSRs and students' EBPs were moderated by (a) culture; (b) age; (d) report types of EBPs; or (e) gender.

Culture was coded as “Eastern,” “Western,” and “other”; “Eastern” referred to students from Asian countries such as China (mainland China, Hong Kong, Taiwan), South Korea, Philippines, Singapore, and so on. “Western” referred to students from European and North American countries such as Germany, the United States of America, and so on. Age was coded as “Kindergarten (3–6 years),” “lower grades of primary school (6–9 years),” “higher grades of primary school (9–12 years),” “Middle school (12–15 years),” “High school (15–18 years),” and “Mixed.” “Mixed” indicated that students included at least two of the above categories. Report type of EBP was coded as “students rated,” “teacher rated,” “peer rated,” “parent rated.” Gender was coded as the proportion of male students.

### Data analysis

All data were analyzed using Comprehensive Meta-Analysis software (CMA Version 2.0). A fixed effects model calculated the homogeneity test and mean effect. Averaged weighted (within- and between-inverse variance weights) correlation coefficients of independent samples were used to compute mean effect sizes. Moderators were decided by the homogeneity test, which revealed variance in effect sizes between different samples' characteristics. When the homogeneity test was significant (*Q*_*Bet*_ > 0.05), *post-hoc* contrasts were implemented to test whether the groups were statistically different. This study used meta-analysis to test whether each moderator accounted for the variation in the effect sizes.

## Results

### Correlation between affective TSRs and students' EBPs

After filtering the literature, we used 57 independent samples and calculated 149 effect sizes (78 effect sizes between positive indicators of affective TSRs and EBPs and 71 effect sizes between negative indicators of affective TSRs and EBPs). In these reviewed studies, 73,933 students participated, and the sample sizes ranged from 20 to 2335.

We calculated sample sizes (*k*), weighted effect sizes (*r*), and 95% confidence intervals (see Table 2). Furthermore, a fixed effects model was used to homogenize the analysis. The results showed significant negative correlations between positive indicators of affective TSRs and EBPs (*r* = −0.263 [*z* = −52.031, *p* < 0.001, *k* = 78, 95% CI = −0.272, −0.253]) and significant positive correlations between negative indicators of affective TSRs and EBPs (*r* = 0.554 [*z* = 118.588, *p* < 0.001, *k* = 71, 95% CI = 0.547, 0.561]). These effect sizes were suitable for moderator analysis.

**Table 2:** **Fixed-model of the correlation between affective TSRs and students' EBPs**.

	***k***	**N**	**Mean *r* effect size**	**95% CI for** ***r***		**Homogeneity test**			**Tau-squared**			**Test of null (two tailed)**
				**LL**	**UL**	**Q(r)**	***p***	**I-squared**	**Tau-squared**	**SE**	**Tau**	***Z*-Value**
PI	78	37375	−0.263	−0.272	−0.253	879.022	0.00	91.126	0.022	0.005	0.149	−52.031^***^
NI	71	36350	0.554	0.547	0.561	2431.398	0.00	97.121	0.067	0.017	0.260	118.588^***^

*** P < 0.001. PI, Positive indicators of affective TSRs, NI, Negative indicators of affective TSRs.

### Moderator analysis

We conducted two total homogeneity tests across 78 (PI) and 71 (NI) independent samples. The results showed a significant homogeneity coefficient between affective TSRs and students' EBPs [*Q*_T__(77)PAE_ = 879.022, *p* < 0.001; *Q*_T__(70)NAE_ = 2431.398, *p* < 0.001]. These results indicate that culture, age, report types of EBPs and gender might moderate the links between affective TSRs and students' EBPs. Therefore, we used meta-analysis of variance to examine whether culture, age, and report types of EBPs moderated the correlations between affective TSRs and students' EBPs, and we used meta-regression analyses to examine whether gender influenced the relation between affective TSRs and students' EBPs.

#### Culture

As indicated in Table 3, a homogeneity test showed a significant homogeneity coefficient between positive indicators of affective TSRs and EBPs across Eastern culture students and across Western culture students (Q_BET_ = 8.816, df = 1, *p* < 0.001). In particular, Table 3 shows that the Western students (*r* = −0.267, 95% CI = −0.277, −0.258) indicated a stronger correlation between positive indicators of affective TSRs and EBPs than the Eastern students (*r* = −0.207, 95% CI = −0.246, −0.167). Likewise, the homogeneity test found significant differences in the correlation between negative indicators of affective TSRs and EBPs across the two cultures (Q_*BET*_ = 25.307, df = 1, *p* < 0.001). Table 3 also shows a stronger correlation between negative indicators of affective TSRs and EBPs among Eastern students (*r* = 0.675, 95% CI = 0.631, 0.714) than Western students (*r* = 0.551, 95% CI = 0.544, 0.558).

**Table 3:** **Culture value, age, and report types of EBPs as moderators of the links between affective TSRs and EBPs**.

	**Between-group effect (Q_BET_)**	***k***	***N***	**Mean *r* effect size**	**SE**	**95% CI for *r***		**Homogeneity test within each group (QW)**
						**LL**	**UL**	
**POSITIVE INDICATORS OF AFFECTIVE TSRs**								
Culture	8.816^**^							
Eastern		6	2283	−0.207	0.021	−0.246	−0.167	52.600^***^
Western		72	35092	−0.267	0.006	−0.277	−0.258	811.002^***^
Age	134.316^***^							
Kindergarten		25	8913	−0.191	0.003	−0.211	−0.171	64.463^***^
Lower grades		17	7441	−0.285	0.022	−0.305	−0.263	353.170^**^
Higher grades		23	8322	−0.227	0.013	−0.247	−0.206	271.401^***^
Middle school		4	1062	−0.247	0.014	−0.303	−0.189	13.145^*^
High school		1	415	−0.280	0.000	−0.366	−0.189	0.000
Mixed		8		−0.333	0.002	−0.350	−0.317	35.922^***^
Report type	101.736^***^							
Teacher		51	22527	−0.284	0.008	−0.296	−0.272	603.690^***^
Self		14	4920	−0.172	0.004	−0.199	−0.145	42.945^***^
Peer		6	3370	−0.172	0.004	−0.204	−0.139	18.422^***^
Parent		7	6558	−0.307	0.020	−0.329	−0.285	105.624^***^
**NEGATIVE INDICATORS OF AFFECTIVE TSRs**								
Culture	25.307^***^							
Eastern		3	660	0.675	0.046	0.631	0.714	17.802^***^
Western		68	35690	0.551	0.017	0.544	0.558	2388.289^***^
Age	178.539^***^							
Kindergarten		31	11330	0.484	0.022	0.470	0.498	694.015^***^
Lower grades		20	9756	0.557	0.030	0.543	0.571	714.126^***^
Higher grades		14	4375	0.619	0.065	0.600	0.637	495.949^***^
Mixed		6	10880	0.591	0.028	0.579	0.603	348.770^***^
Report type	349.373^***^							
Teacher		58	26321	0.602	0.019	0.594	0.610	1771.999^***^
Self		5	1202	0.314	0.092	0.262	0.364	81.845^***^
Peer		2	1001	0.404	0.010	0.351	0.455	3.303
Parent		5	7256	0.429	0.008	0.410	0.448	53.868^***^
others		1	570	0.337	0.000	0.262	0.408	0.000

* p < 0.05,

** p < 0.01,

*** p < 0.001.

Lower grades, lower grades of primary school; higher grades, higher grades of primary school.

#### Age

The results of the homogeneity test (Q_BET_ = 134.316, df = 5, *p* < 0.001) suggested that the link between affective TSRs and EBPs was influenced by age. Positive indicators of affective TSRs were negatively related to EBPs for kindergarteners (*r* = −0.191, 95% CI = −0.211, −0.171), LPG students (*r* = −0.285, 95% CI = −0.305, −0.263), HPG students (*r* = −0.227, 95% CI = −0.247, −0.206), middle school students (*r* = −0.247, 95% CI = −0.303, −0.189), high school students (*r* = −0.280, 95% CI = −0.366, −0.189), and mixed students (*r* = −0.333, 95% CI = −0.350, −0.317). Results indicate that the correlation between positive indicators of affective TSRs and EBPs was stronger among LPG students than other students (except mixed group) and weaker among kindergarten students than other students. As shown in Table 3, the homogeneity test (Q_BET_ = 178.539, df = 3, *p* < 0.001) suggested that age moderated the link between negative indicators of affective TSRs and EBPs. Negative indicators of affective TSRs were positively linked to EBPs for kindergarteners (*r* = 0.484, 95% CI = 0.470, 0.498), LPG students (*r* = 0.557, 95% CI = 0.543, 0.571), HPG students (*r* = 0.619, 95% CI = 0.600, 0.637), and mixed (*r* = 0.591, 95% CI = 0.579, 0.603) groups. These results suggest that the correlation between negative indicators of affective TSRs and EBPs increases with age.

#### Report type of EBPs

The results of the homogeneity test (Q_BET_ = 101.736, df = 3, *p* < 0.001) suggested that age influenced the link between affective TSRs and EBPs. Positive indicators of affective TSRs were negatively correlated with EBPs when rated by teachers (*r* = −0.284, 95% CI = −0.296, −0.272), students (*r* = −0.172, 95% CI = −0.199, −0.145), peers (*r* = −0.172, 95% CI = −0.204, −0.139), or parents (*r* = −0.307, 95% CI = −0.329, −0.285). The correlation between positive indicators of affective TSRs and EBPs were stronger when rated by teachers or parents than by others. As shown in Table 3, the homogeneity test (Q_BET_ = 349.373, df = 4, *p* < 0.001) suggested that age moderated the link between negative indicators of affective TSRs and EBPs. Negative indicators of affective TSRs were positively correlated to EBPs when rated by teachers (*r* = 0.602, 95% CI = 0.594, 0.610), students (*r* = 0.314, 95% CI = 0.262, 0.364), peers (*r* = 0.404, 95% CI = 0.351, 0.455), or parents (*r* = 0.429, 95% CI = 0.410, 0.448). These results indicate that the correlation between negative indicators of affective TSRs and EBPs were lower when student rated than when rated by others.

#### Gender

To examine whether gender moderated the links between affective TSRs and students' EBPs, r was meta-regressed onto the percentage of male students in each sample. In Table 4, meta-regression analysis (Q _Model_ [1, *k* = 74]_NI_ = 4.106, *p* < 0.05) showed that gender moderated the link between positive indicators of affective TSRs and students' EBPs; as the proportion of female students increased, the link was stronger. The correlations between positive indicators of affective TSRs and EBPs for an all-female sample (*r* = −0.315) were stronger than those for an all-male sample (*r* = −0.249). In contrast, meta-regression analysis (Q _Model_[1, *k* = 66] _PAE_ = 1.666, *p* > 0.05) showed that gender did not moderate the link between negative indicators of affective TSRs and students' EBPs.

**Table 4:** **Meta-regression analyses with effect size regressed onto percentage of male students**.

	**Variables**	**Parameter**	**Estimate**	**SE**	***Z*-value**	**95%CI for b**	
						**LL**	**UL**
Positive indicators	Male (%)	β_0_	−0.315	0.017	−18.313	−0.349	−0.281
		β_1_	0.066	0.033	2.026	0.002	0.130
		Q _Model_(1, *k* = 74) = 0.4.106, *P* < 0.05					
Negative indicators	Male (%)	β_0_	0.678	0.022	30.266	0.634	0.722
		β_1_	−0.0563	0.044	−1.291	−0.014	−0.029
		Q _Model_(1, *k* = 66) = 1.666, *P* > 0.05					

### Publication bias

**Figure 1:**
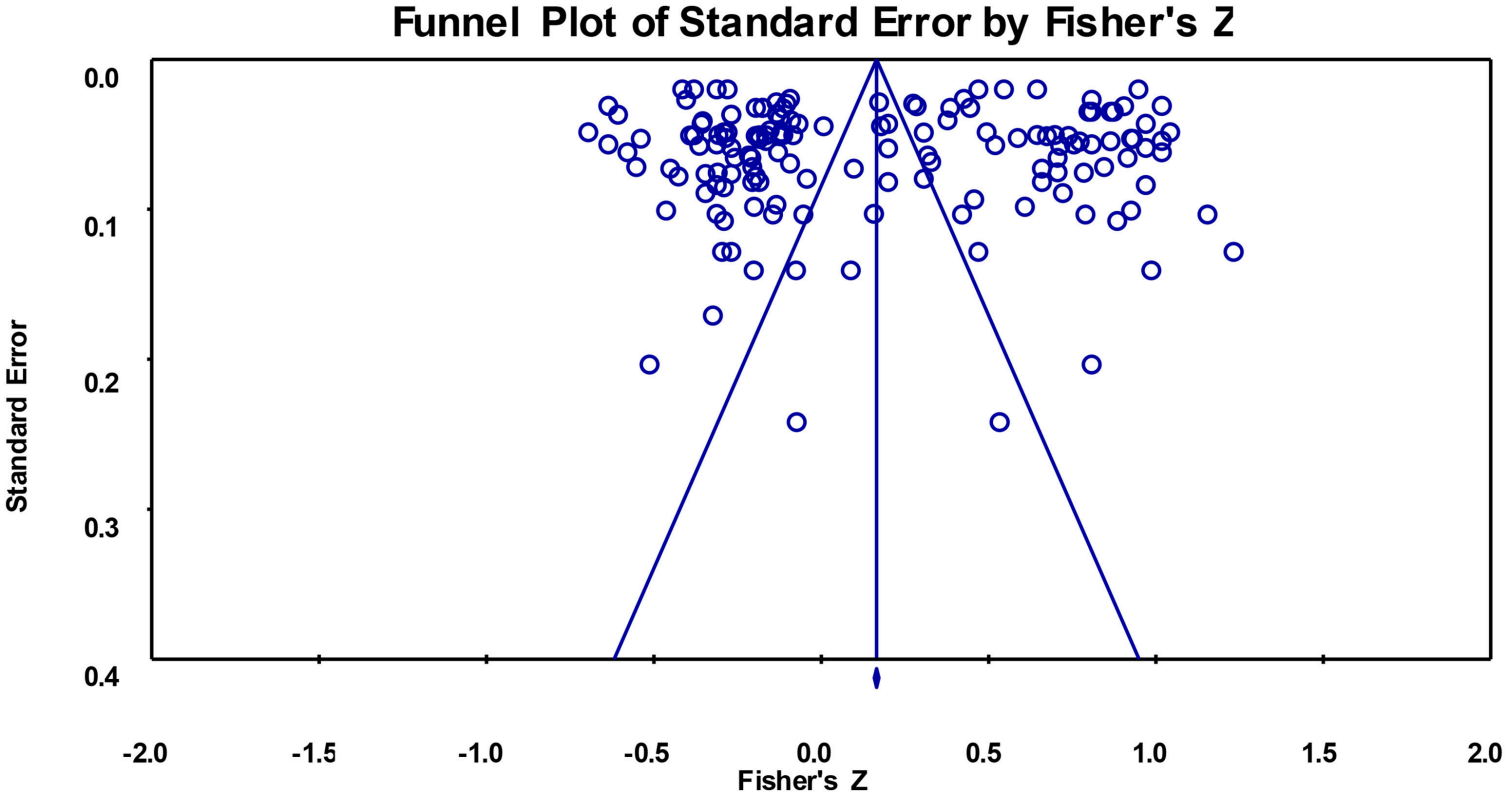
**Funnel plot of effect sizes of the correlation between affective teacher-student relationships and students' externalizing behavior problems**.

To examine whether the results were biased due to effect sizes from various sources, we drew a funnel plot (see Figure 1). It showed that the 149 effect sizes were symmetrically distributed on both sides of the average effect size, and an Egger's regression (Egger et al., 1997) revealed no significant bias [*t*_(147)_ = 0.010, *p* = 0.991 > 0.05]. Egger's regression is an effective method for examining publication bias (Teng et al., 2015). In addition, we conducted Egger's regression analysis on both positive and negative indicators of affective TSRs and EBPs. The results also showed no publication bias [*t*_*PI*__(76)_ = 0.767, *p* = 0.445 > 0.05; *t*_*NI*__(69)_ = 0.568, *p* = 0.572 > 0.05]. Together, these results indicated stability in the overall correlation between affective TSRs and students' EBPs in this study.

## Discussion

In the current meta-analysis 57 recent studies, with 149 effect sizes and 73,933 students are reviewed. We examined the effect sizes of correlations between affective TSRs and students' EBPs, revealing culture, age, report type of EBPs and gender as moderators influencing the links. The results showed that negative affective TSRs was negatively correlated with students' EBPs and negative affective TSRs was positively correlated with students' EBPs. The correlation coefficients for these results were both medium. In addition, these results showed that students' cultures, ages, genders, and report type of EBPs moderated the link between affective TSRs and students' EBPs.

### The significant correlation between affective TSRs and students' EBPs

The meta-analysis results indicated a significant negative correlation between positive indicators of affective TSRs and EBPs and a significant positive correlation between negative indicators of affective TSRs and EBPs. These results suggested that affective TSRs help students reduce EBPs. As indicated by Masten and Garmezy (1985), TSRs are an important support system for students' behavioral development and many studies focusing on improving students' behavior problems with TSRs. Moreover, students with closer TSRs had fewer antisocial behaviors (Birch and Ladd, 1998), and high levels of TSR closeness outperformed students' early problem behaviors when predicting their later behavior problems (Pianta and Nimetz, 1991). In addition, this study found that, compared with the positive indicators of affective TSRs, negative indicators of affective TSRs showed more strong correlation with students' EBPs, suggesting that negative affect TSRs are more influential than positive affect TSRs on students' EBPs. These results suggest that reducing negative affective TSRs or increasing positive affective TSRs might reduce individuals' EBPs. Therefore, teachers might explore using diverse communication strategies to help students build positive affective TSRs and reduce negative affective TSRs. In addition, results suggest that targeted interventions might help students develop affective TSRs when they show EBPs.

This study's results support the direct effect model but did not test the indirect effect model. Future studies can test the indirect effect model of affective TSRs and students' EBPs.

### Moderating effects

Moderation analysis showed that students' cultures, ages, genders, and the report type of EBPs moderated many of the links between affective TSRs and students' EBPs. Gender did not moderate the link between the negative indicators of affective TSRs and EBPs.

#### Moderating role of culture

We hypothesized that students' culture might moderate the link between affective TSRs and students' EBPs. The results of this meta-analysis support this hypothesis. In particular, the correlation between positive indicators of affective TSRs and students' EBPs was stronger among Western students than Eastern ones. In contrast, the correlation between negative indicators of affective TSRs and students' EBPs was stronger among Eastern students than Western ones. These results suggest that positive affective TSRs might reduce EBPs more for Western students than for Eastern ones. In contrast, negative affective TSRs might increase EBPs more for Eastern students than Western ones. These results are consistent with previous studies (Fowler et al., 2008; Solheim et al., 2011; Zhang and Sun, 2011). The higher expectations and stricter TSRs in collectivist Eastern cultures compared to the lower expectations of relaxed TSRs in individualistic Western cultures might account for these differences; positive, relaxed TSRs might cultivate good behaviors and limit behavior problems while negative, strict TSRs might yield behavior problems more easily. These results suggest that differences in students' cultures must be considered when developing affective TSRs to reduce students' EBPs. Together, they suggest that training and interventions based on the specific culture of the student might be beneficial.

#### Moderating role of age

This meta-analysis found that age moderates the link between affective TSRs and students' EBP, consistent with past studies (Hughes and Cavell, 1999; Denham et al., 2000). Furthermore, additional analysis found that LPG students showed a stronger correlation between positive indicators of affective TSRs and EBPs than those in kindergarten and HPG students. Students' developing emotions at these ages and their interest in talking and building relationships with their teachers might explain these differences. LPG students might be exploring emotional relationships with their teacher and hence might be more willing to listen to their teachers' suggestions about correcting their EBPs. Additional analysis showed that the correlation between negative indicators of affective TSRs and EBPs are stronger among HPG students than kindergarteners or LPG students, possibly because as students get older, the proportion of positive affective TSRs decreases while that of negative affective TSRs increases (Wang and Wang, 2002). LPG students might be more likely than younger students to use disruptive behaviors to attract teacher attention; these behaviors can reduce the positive affect and increase the negative affect of their TSR, fostering a vicious cycle between affective TSRs and students' EBPs. These results suggest that we pay closer attention to the age and development of students when developing affective TSRs to reduce students' EBPs.

#### Moderating role of report type of EBPs

This study showed that the report type of EBPs moderates the link between affective TSRs and students' EBPs. Specifically, the correlation between positive indicators of affective TSRs and EBPs were stronger when rated by teachers or parents than by others. Also, the correlation between negative indicators of affective TSRs and EBPs was lower when rated by students or peers than otherwise. These results are supported by many other studies (Loeber et al., 1990; Deater-Deckard et al., 1998) and suggest that a link between affective TSRs and students' EBPs is more visible when rated by teachers or parents than otherwise. Teachers and parents might exaggerate the degree of the link between affective TSRs and students' EBPs, if students and peers understand their own EBPs better than their teachers and parents do (Achenbach, 1991). A possible alternate explanation is that students and peers downplay this link, as they pay less attention than teachers or parents to the teacher's role in students' behavior development.

#### Moderating role of gender

This study showed that gender moderates the links between positive affective TSRs and students' EBPs. As predicted, the all-female group showed a stronger correlation between positive indicators of affective TSRs and students' EBPs than the all-male group. However, gender did not moderate the links between negative indicators of affective TSRs and students' EBPs. These results suggest that positive affective TSRs reduce female students' EBPs more easily than they do those of male students, possibly because female students care more about their relationships with their teachers, seek more positive emotions from them (Hu et al., 2015), and are more easily influenced by them, resulting in fewer EBPs compared to male students (Deater-Deckard and Dodge, 1997). In addition, this result suggest that we might need to attend more to developing TSRs with male students than with female students to reduce their EBPs.

### Limitations and implications

This meta-analysis has several limitations. First, only closeness, warmth, support, empathy, trust, sensitivity, conflict, negativity, and anger were selected as indicators of affective TSRs; other indicators, such as concern, caring, were not found. Furthermore, the selected indicators may overlap. Second, this study selected several familiar indicators of EBPs; others indicators, such destructive behavior, were excluded. Third, all the studies reviewed examined only direct effects; however, other studies have found that affective TSRs affects students' EBPs across other variables as well (Stanger and Lewis, 1993; Yoon, 2002). Therefore, future studies should test the indirect effects of affective TSRs on students' EBPs. Fourth, this study only considers whether students' culture, age, gender, and report type of EBPs moderated the link between affective TSRs and students' EBPs. Other variables, notably other indicators of affective TSRs and students' EBPs, should be examined in future studies as they may influence the links between affective TSRs and students' EBPs. Fifth, this study included only English articles, which may have narrowed its scope and neglected some cultures. Sixth, this meta-analysis was based on cross-sectional studies and correlational data; hence a causal relationship cannot be inferred.

## Conclusion

Through reviewing 57 studies, 149 effect sizes, and 73,933 student participants, meta-analysis results revealed that positive and negative affective TSRs were significant correlated with students' EBPs. Furthermore, these correlations were moderated by students' culture, age, report type of EBPs, and gender. In particular, negative affective TSRs were more strongly linked than positive affective TSRs to students' EBPs. Also, the negative correlation between positive indicators of affective TSRs and EBPs was stronger among Western students than Eastern students. In contrast, the positive correlation between negative indicators of affective TSRs and EBPs was stronger among Eastern students than Western students. The negative correlation between positive indicators of affective TSRs and EBPs was stronger among LPG students than among other students (except the mixed group). Also, the positive correlation between negative indicators of affective TSRs and students' EBPs was stronger among HPG students than other students. The negative correlation between positive indicators of affective TSRs and EBPs was stronger when rated by teachers or parents than by students or peers. However, the positive correlation between negative indicators of affective TSRs and EBPs was stronger when rated by students or peers. The negative correlation between positive indicators of affective TSRs and students' EBPs was stronger among girls than among boys. However, gender did not moderate the link between negative indicators of affective TSRs and students' EBPs. This meta-analysis estimated effect sizes for students' EBPs during the past 17 years and suggests that differences in students' cultures, age, and gender can inform future research and practices.

## Author contributions

HL and YC provided the idea, designed this study and wrote the manuscript. HL contributed to data analysis and data collection. MC contributed to paper writing. All authors read and approved the manuscript.

## Funding

This research was supported by the 2015 Excellent Doctoral Training Program of East China Normal University (PY2015003), and the Humanities and Social Sciences Key Project of the Ministry of Education in China (11JJD880003).

### Conflict of interest statement

The authors declare that the research was conducted in the absence of any commercial or financial relationships that could be construed as a potential conflict of interest.
